# The Neutrophil-to-Lymphocyte Ratio as a Prognostic Biomarker of Fournier’s Gangrene Severity: A Meta-Analysis

**DOI:** 10.3390/idr17030055

**Published:** 2025-05-15

**Authors:** Konstantinos Seretis, Nikolaos Bounas, Konstantinos Sfaelos, Georgios Gaitanis, Ioannis Bassukas

**Affiliations:** 1 Department of Plastic and Reconstructive Surgery, Faculty of Medicine, School of Health Sciences, University of Ioannina, 45100 Ioannina, Greece; bounasnikos@gmail.com; 2 Department of Skin and Venereal Diseases, Faculty of Medicine, School of Health Sciences, University of Ioannina, 45100 Ioannina, Greece; k.sfaelos@uoi.gr (K.S.); ggaitan@uoi.gr (G.G.); ibassuka@uoi.gr (I.B.)

**Keywords:** Fournier gangrene, complete blood count, biomarkers, morbidity

## Abstract

Background/Objectives: Fournier’s Gangrene (FG) is a severe and potentially fatal necrotizing infection of the perianal and genital regions, which necessitates prompt therapeutic interventions to prevent disease progression. Accruing evidence from recent research indicates that the neutrophil‒to-lymphocyte ratio (NLR) can predict clinical severity and mortality risk in patients with critical illnesses across various etiologies. This meta-analysis aimed to assess the efficacy of NLR as a prognostic indicator for mortality in patients with FG. Methods: An electronic literature search was conducted across several databases from their inception to 31 May 2024, following a predetermined protocol. Study quality was evaluated using the Cochrane risk of bias tool. A random-effect model was utilized to synthesize the available data. Results: Twelve studies reporting on 767 patients were included in the meta-analysis. Higher NLR levels at presentation were recorded in non-survivors than in survivors (MD = 4.49 [95% CI: 0.67–8.32]; *p* = 0.02). A 76% increased mortality risk was detected for patients with an NLR ≥ 8 (1.76 RR [1.35–2.3], *p* = 0.0001), and the mortality risk was more than twofold greater for patients with an NLR ≥ 10 compared to the remaining patients (RR = 2.31 [1.27–4.21], *p* = 0.006). All included studies exhibited a moderate to serious risk of bias. Conclusions: This meta-analysis reveals that the NLR represents a promising biomarker that can serve as a prognostic indicator in patients with FG. Future studies should address the establishment of proper disease-specific cutoff values to aid in clinical decision-making.

## 1. Introduction

Fournier’s gangrene (FG) is an uncommon, rapidly progressing, and potentially fatal necrotizing skin and soft tissue infection, characterized by polymicrobial involvement (aerobic/anaerobic and gram-negative/-positive) that involves the external genitalia and perineal or perianal regions [1,2]. FG affects both sexes; however, most patients are male with a distinct comorbidity profile that predisposes them to invasive bacterial diseases and disturbed tissue blood perfusion, such as a history of diabetes mellitus, malignancy, and certain neurologic diseases. Within the framework of a crucial interplay between infectious-toxic and tissue damage–associated pathophysiological deviations, the initially spatially confined condition rapidly progresses into a state of an aggravated systemic inflammatory response, which, in some patients with FG, may herald a nonreversible, sepsis-triggered multiorgan failure syndrome. The timely onset of appropriate therapeutic interventions, consisting of comprehensive surgical debridement, fascia incision, removal of necrotic tissue, and appropriate antibiotic therapy under intensive care conditions, is a crucial measure for preventing the development of sepsis and multiorgan function failure, the main immediate causes of mortality in patients with FG [3,4]. Despite the significant progress in FG management, the disease-specific fatality rate remains high, at approximately 10%, even among patients admitted to specialized centers [3,5].

From a clinical perspective, the assessment of severity is crucial for planning a multidisciplinary therapeutic approach for a particular patient. Abnormal clinical and laboratory parameters (fever, tachycardia, and tachypnea; and elevated potassium, sodium, creatinine, leukocyte, and bicarbonate levels) are frequently present at admission. Based on the synthesis of these parameters, various scoring systems have been designed to stratify disease severity and predict mortality in individuals with FG [6,7]. The prototype of the prognostic instruments is the Fournier gangrene severity index (FGSI), which was proposed in 1995 and has been repeatedly validated thereafter [8]. Currently, the determination of FGSI scores (or their subsequent modifications, Simplified Fournier’s Gangrene Severity Index (SFGSI) and Uludag Fournier’s Gangrene Severity Index (UFGSI)) is a part of the routine clinical evaluation during the admission of patients with FG [9,10]. In addition to the specialized FG indices above, the Laboratory Risk Indicator for Necrotizing Fasciitis (LRINEC) score has also been applied to evaluate the severity of patients with FG [11].

Accumulating evidence over the last decade highlights the neutrophil-to-lymphocyte ratio (NLR) as a rather ‘agnostic’ indicator of clinical severity and a correlator of mortality risk in patients with underlying pathological conditions, including inflammatory diseases, systemic infections, malignancies, and patients with all-cause critical illnesses [12,13]. The NLR, the quotient of the neutrophil to lymphocyte counts in peripheral blood, is a simple laboratory value that integrates core proxy information on the interplay between innate and adaptive immune responses [13,14]. Alterations in the NLR reflect shifts in the ratio between neutrophil and lymphocyte counts, both of which display increases in a different manner depending on the type of pathogen involved (e.g., virus and bacteria) [14,15]. In healthy individuals, NLR values typically range between 1 and 3, reflecting a balanced immune response [16]. Mild elevations (NLR between 3 and 5) may be associated with physiological stress, low-grade inflammation, or early systemic responses [17]. NLR values exceeding 5 are often indicative of more pronounced systemic inflammation, while values greater than 10 are commonly suggestive of sepsis or critical illness [16,17]. Neutrophils play a crucial role as primary mediators of the innate immune response [18]. On the other hand, lymphocytes represent the activity of the adaptive immune system, and large cohort studies have demonstrated that lymphopenia is linked to an increased risk of all-cause mortality [19,20]. Finally, from a practical point of view, the NLR is a convenient, readily available, and low-cost parameter, which makes it a valuable biomarker in clinical settings. A notable advantage of the NLR is that, in most cases, it appears to offer greater reliability than either the neutrophil or the lymphocyte counts alone when predicting patient survival [21,22,23]. Although several confounders, including medications and comorbidities, can influence NLR values, the NLR is established as a useful prognostic indicator for a wide spectrum of clinical conditions, including patients with critical care infectious disease [24]. Interestingly, the NLR also seems to be a strong predictor of all-cause mortality risk in cohorts of previously healthy individuals [25,26]

A recent meta-analysis underlined the validity of the FGSI and the combined UFGSI and SFGSI score calculations in predicting the mortality risk of patients with FG [7]. Additionally, the NLR, since its introduction as a disease severity index for patients with FG, has been evaluated as a potential predictor of the risk of intensive care admission, the need for mechanical ventilation, and the mortality of patients with FG [27]. Herewith, we present the results of a systematic review and meta-analysis of the NLR as a prognostic indicator of FG mortality.

## 2. Materials and Methods

We established a predefined protocol, following the recommendations outlined in the Cochrane Handbook, which was registered in the PROSPERO database (registration number: CRD42024524979) [28]. The meta-analysis complied with the latest PRISMA (Preferred Reporting Items for Systematic Reviews and Meta-Analyses) guidelines, as detailed in Table S1 (Supplementary Material) [29]. Patient consent forms and ethics committee approval were not deemed applicable, as this study is a systematic review article.

### 2.1. Search Strategy

An electronic literature search was conducted across the MEDLINE (PubMed), Scopus, Cochrane Library, and CENTRAL databases from inception up to 31 May 2024. A complementary search, using Google Scholar, was performed with the same predefined keywords to identify any additional relevant studies not captured in the initial database search. The search terms “neutrophil-to-lymphocyte” and “Fournier gangrene” were applied, limited to the ‘Title’ and ‘Abstract’ fields. No restrictions on time or language were imposed. The strategy is provided in Appendix A. To enhance search sensitivity and uncover additional relevant studies, the references of the retrieved articles were also reviewed.

### 2.2. Eligibility of Relevant Studies and Study Selection

Studies were included based on the following criteria: (1) reporting the NLR; (2) providing data on FG; and (3) being published in a peer-reviewed journal. Exclusion criteria comprised studies focusing on infections other than FG and those that did not report NLR values in FG patients. Additionally, reviews, duplicate publications, editorials, and studies involving nonhuman subjects were excluded.

Two reviewers (K.S. and N.B.) conducted the literature search and screened the retrieved records independently in a blinded manner, along with the full texts of potentially eligible studies, for relevance. Disagreements were resolved through mutual agreement.

### 2.3. Data Collection and Risk of Bias Assessment

Data extraction was carried out by the same two reviewers independently via a standardized form. Any discrepancies were resolved through mutual agreement. We collected data on general study characteristics, patient demographics, and relevant outcomes. The primary outcome was the value of the peripheral blood NLR, which could serve as a prognostic or diagnostic biomarker for FG. The NLR was uniformly derived from peripheral venous blood samples collected at admission and calculated as the ratio of the absolute neutrophil count to the absolute lymphocyte count, based on standard complete blood count measurements.

The quality of the included studies was evaluated using the Cochrane risk of bias tool (ROBINS-I) for nonrandomized comparative studies [30].

### 2.4. Data Synthesis and Analysis

A meta-analysis was performed when data were available from at least two studies. For continuous variables (NLR), mean differences (MDs) with 95% confidence intervals (CIs) were calculated, whereas risk ratios (RRs) with 95% CIs were determined for dichotomous outcomes (patients above the cutoff value). An inverse variance statistical approach was applied for continuous variables, and the Mantel‒Haenszel model was used for dichotomous variables. Due to significant heterogeneity in the study designs and sampling, a random effects model was employed for the analyses. A significance level of *p* ≤ 0.05 was set. Sensitivity analyses were also performed to explore potential sources of heterogeneity among the studies. Heterogeneity was assessed through Cochran’s Q test and Higgins’ I^2^ statistic. Forest plots were created to display the effect sizes of each study along with the corresponding 95% CIs. Publication bias was assessed through Funnel plots. Egger’s test was performed if the number of studies analyzed allowed for its calculation, ensuring adequate statistical power. Spearman correlation coefficient was utilized, due to the non-normal distribution of the data, to assess the presence of collinearity between NLR values and age variables in both study groups, along with the formation of scatter plots for visual examination. The meta-analysis was conducted via the ‘meta’ package in R, version 4.2.3 (R Foundation for Statistical Computing, Vienna, Austria) [31].

## 3. Results

**Figure 1:**
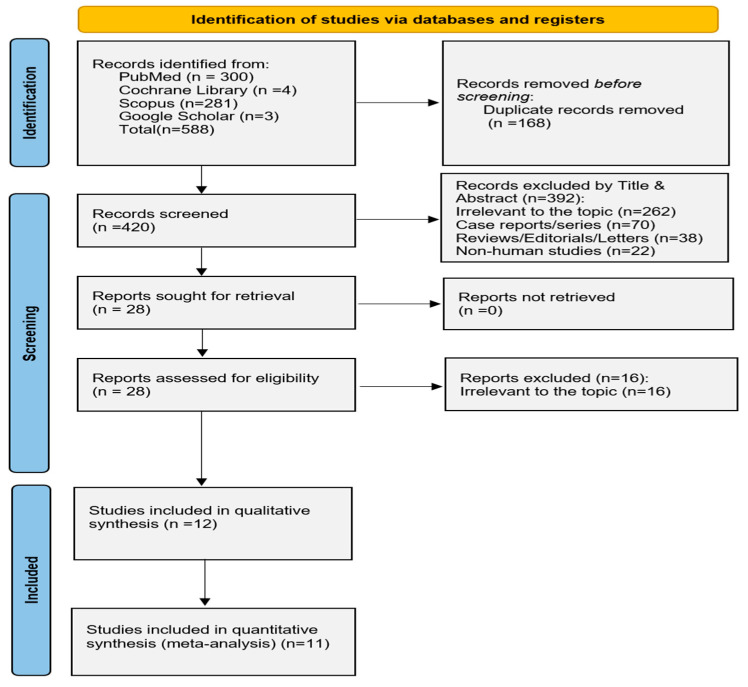
PRISMA flowchart.

The process of study selection is outlined in Figure 1. Out of 588 records, 12 studies fulfilled the inclusion criteria and were thus incorporated into the data synthesis and subsequent data analysis models [32,33,34,35,36,37,38,39,40,41,42,43].

### 3.1. General Study Characteristics

The 12 included studies were carried out in Turkey (=6), South Korea (=2), Indonesia (=2), China (=1), and Switzerland (=1). All studies were published between 2015 and 2023. They were observational studies of retrospective design, studying a total of 767 patients (Table 1). Blood samples taken at patient admission were used to calculate the NLR values.

The risk of bias was deemed moderate to serious, based on the study's quality, as indicated in Supplementary Digital Content Tables showcasing the ROBINS-I quality assessments (Table S2 and Figure S1). Publication bias was evaluated through visual examination of the funnel plots for all analyses, which consistently demonstrated relative symmetry (Figure S2, Figure S3, Figure S4, Figure S5, Figure S6 and Figure S7). Egger’s test was conducted only for the NLR difference outcome, as applying it to the other outcomes would have been statistically underpowered due to the limited number of studies. The intercept was −0.83 [−2.17: 0.5], t = −1.22, and *p* = 0.26.

### 3.2. Patient Characteristics and Baseline Clinical Profile

A total of 767 patients were included in the meta-analysis, with 606 patients comprising the survivor group and 161 in the non-survivor group. The baseline characteristics of the individuals in the studies are provided in Table 1. The mean mortality rate (MR) was 0.2816 (95% CI: 0.2218–0.3503), with a rather high degree of heterogeneity (I^2^ = 58.72%; 95% CI: 21.87–78.19). A sex comparison between the two groups, including data from seven studies, indicated a marginally higher, though statistically not significant, mortality risk among female patients (OR = 1.1363; 95% CI: 0.8869–1.4559). Twelve studies reported the age of the patients, with a MD of 10.05; a significant difference was observed between the two groups (*p* < 0.001). Nine studies reported a diagnosis of diabetes mellitus, but no significant difference was found between the groups (OR = 1.37, *p* = 0.28). Lastly, three studies presented data on hypertension, but no significant difference was identified (OR = 1.12, *p* = 0.72).

### 3.3. NLR as a Prognosticator Index

**Figure 2:**
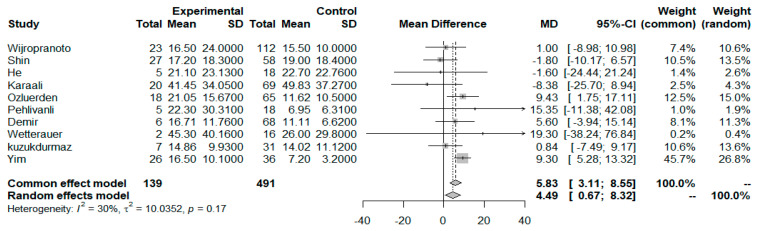
NLR mean difference forest plot for survivors vs. non-Survivors.

A total of 10 studies reported the difference in the NLR between non-survivors and survivors of FG, providing data for 630 patients, 491 in the survivor and 139 in the non-survivor group. The NLR at presentation was significantly greater in the non-survivors than in the survivors (MD = 4.49 [95% CI: 0.67–8.32]; *p* = 0.02), with only mild heterogeneity present in the analysis model (I^2^ = 30.1%, *p* = 0.17) (Figure 2). Further examination of the NLR distribution in relation to the patients’ age indicated that for this specific group of patients, age did not correlate with the NLR values for either survivors or non-survivors, as shown in the produced scatter plots for the NLR–Age variables of the two groups (Figure S8 and Figure S9). Further analysis yielded a Spearman correlation coefficient of −0.36 (*p* = 0.296) for the survivors and 0.52 (*p* = 0.12) for the non-survivors, verifying the absence of correlation.

**Figure 3:**
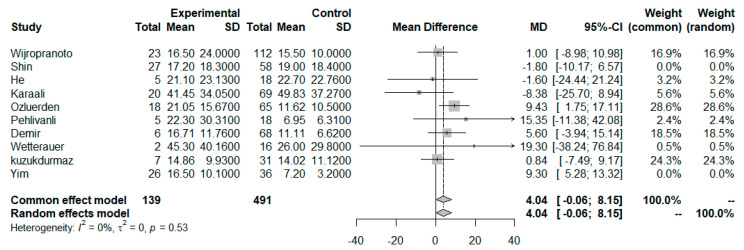
Sensitivity analysis of the NLR mean difference: forest plot for survivors vs. non-survivors.

We proceeded with a sensitivity analysis to explore any potential sources of excess heterogeneity and excluded the studies by Shin et al. [33] and Yim et al. [42], which both exerted an asymmetric high influence on the total effect size. After that, the overall heterogeneity of the studies decreased to zero (I^2^ = 0%, Q statistic *p* = 0.53); however, the NLR values of the non-survivors, compared to those of the surviving patients, were borderline insignificant (MD = 4.04 [95% CI: −0.06–8.15]; *p* = 0.053); (Figure 3). Also, we conducted a sensitivity analysis excluding the studies exhibiting at least one high-risk of bias domain on the ROBINS-I tool. Yet again, the NLR mean difference remained statistically significant between the two groups, a result that further enhances the credibility of the reported outcomes (MD = 4.67 [0.30–9.03]; *p* = 0.036, two-tailed test, I^2^ = 35.7%), as shown in the forest plot in Fig. SDC 12 (Figure S10). Finally, subgroup analysis by country of study origin revealed no significant differences (Q = 0.88, *p* = 0.927), as indicated in the plots shown in Fig. SDC 13 (Figure S11).

### 3.4. NLR Cutoff Values and FG Prognosis

**Figure 4:**
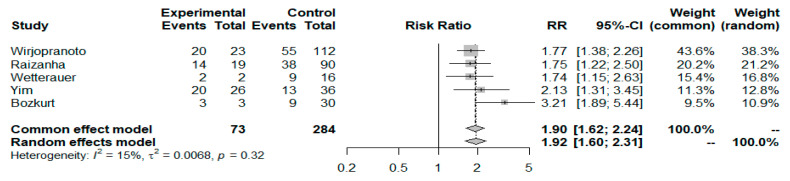
Forest plot of NLR cut-off values and FG mortality.

Five studies, including 357 patients (73 non-survivors vs. 284 survivors), investigated the application of cutoff levels of the NLR to predict patient mortality. The synthesis of these data revealed a 92% increased mortality risk for patients with NLR values at admission over the arbitrarily by the authors’ specified NLR cut-off thresholds of 8 and 10 (risk ratio: 1.92; 95% CI: 1.60–2.31; I^2^ = 15.3%, *p* < 0.001) (Figure 4). Further analysis of the available data revealed that Yim et al. [42], Wirjopranoto et al. [32], and Wetterauer et al. [40] evaluated the prognostic impact of a cutoff value of the NLR of 8. The meta-analysis of these three studies (involving 216 patients, 164 survivors and 52 non-survivors) indicated that patients with an NLR ≥ 8 had a 76% increased risk of disease-specific mortality compared with patients with an NLR < 8 (RR = 1.76; 95% CI: 1.35–2.30; I^2^ = 17.4%, *p* < 0.001), as shown in the results of the forest plot (Figure S12). Additionally, Wetterauer et al. [40] and Bozkurt et al. [43] studied the effect of setting the NLR cutoff value at 10 as a mortality prognosticator for FG patients. The meta-analysis of these two small studies (51 included patients in total, 46 survivors and 5 non-survivors) highlighted a greater than twofold mortality risk for patients with an NLR ≥ 10 compared to the remaining patients (RR = 2.31 [95% CI: 1.27–4.21], I^2^ = 69%, *p* = 0.006], with the results shown in the forest plot (Figure S13). Finally, in a cohort study that included 109 patients with FG, Raizandha et al. applied a receiver operating characteristic (ROC) approach to assess the cutoff NLR for patient survival, which was 10.9 [34]. Univariate Kaplan‒Meier survival analysis, with this cutoff value used to allocate the patients with FG into two NLR groups, showed that the NLR can serve as an independent predictor of mortality (HR = 5.18 [95% CI: 1.09–8.47], *p* < 0.05).

## 4. Discussion

The main objective of this review was to provide a synthesis of the pertinent literature, focusing on the relationship between NLR values at admission and the subsequent disease-specific mortality risk in patients with FG. The results clearly indicate that, compared with the surviving patients, the patients with FG who did not survive had, on average, significantly higher numerical NLR values. Additionally, data derived from studies that evaluated patient outcomes according to a researcher-determined NLR cutoff value reached the same conclusion, with higher cutoffs (cutoff = 10 vs. cutoff = 8) indicating an increased risk of mortality. These findings support the correlation between elevated NLR values and poorer prognosis.

Interestingly, in a retrospective analysis of 68 patients with FG who did not fulfill the inclusion criteria, Kahramanca et al. employed a NLR cutoff value determined via ROC analysis and reported significantly higher NLR values in patients with more severe disease, as determined by the number of surgical debridement rounds (*p* < 0.001) [27]. Moreover, there was no significant difference in the FGSI score between the two groups above, indicating the superiority of the NLR over the FGSI as a marker with prognostic value for patients with FG [27].

At this point, we would like to comment on the position of the NLR in the landscape of the predictors of the severity and clinical outcome of patients with FG, mainly in comparison with the FGSI. Although isolated studies have concluded that the NLR may be marginally superior to the FSGI as an FG prognosticator, the FGSI, including its modifications, is adequately validated and widely accepted as a clinical index in routine practice [6,7]. Given that, in many different settings, the NLR has proven to be a superior clinical indicator compared to either leukocyte or neutrophil counts, it would be worthwhile to address the efficiency of a modified FSGI score that would include the corresponding NLR value in the calculation formula instead of the parameter ‘leukocyte count’ [21,22,23]. Notably, some studies have reported a lack of correlation between the FSGI and the NLR [39,44].

This review, which is based on the principles of meta-analysis, systematically summarizes the available evidence regarding the role of the NLR as a prognostic factor in FG patients at admission. Among its strengths is the methodology employed, which mitigates the risk of bias and improves the overall quality of the evidence analyzed. Assessing the confidence in the reported outcomes, by means of a relevant tool, further improved these meta-analysis findings. Moreover, with respect to disease-specific mortality, the baseline patients’ characteristics analyzed in this review are largely representative of the population of patients with FG. Notably, in the present meta-analysis, in contrast to other studies, neither diabetes mellitus nor hypertension was significantly associated with an increased risk of non-survival [45,46]. Females have a lower incidence of FG compared to males, but they experience a higher mortality rate [47]. The study from Sorensen et al. highlights a higher mortality rate in women, around 20–50%, whereas in males, it amounts to 7.5% [48]. Czymek et al. also concurred that females displayed higher mortality rates, while similar outcomes were reported by a recent study from Spain (OR 1.32 [1.07–1.63] compared to their male counterparts) and by Abbasi et al. (7.1% vs 5.7% in male patients, *p* < 0.0001) [49,50,51]. Finally, the fact that patients’ age was significantly greater in the non-survivor group could have confounded our results, since there is evidence in the literature that NLR values increase with age [52]. However, the inspection of our corresponding plots herein suggests a lack of correlation between these two variables in the present sample of patients with FG, an observation in favor of the plausibility of the currently observed relationship between NLR values and mortality.

This meta-analysis has several limitations. The core limitation is the rather small number of included studies, especially for extensive subgroup analyses. This is more evident in the evaluation of predictive efficacy using the authors’ determined nonuniformly accepted NLR cutoff values to group the included studies, which weakened the strength of the reported results owing to the small sample size. Additionally, the quality assessment tools utilized yielded rather moderate results for the included studies. Furthermore, the retrospective design of the included studies is prone to bias, especially selection and recall bias, although this was less pronounced since data were obtained from hospital records rather than direct patient interviews. Also, the included studies spanned diverse regions and healthcare settings, which could contribute to the observed heterogeneity. Finally, although no significant publication bias was observed, some degree of latent publication bias may be inevitable, as most included studies tended to report positive outcomes.

Overall, further research is needed to deepen our knowledge regarding FG pathogenesis and the underlying molecular interactions and mechanisms involved. This will facilitate the integration of hematological biomarkers, such as the NLR, into daily clinical practice. The incorporation of more clinical data on these biomarkers is expected to enhance their diagnostic accuracy and predictive precision, ultimately guiding clinical decision-making toward a more personalized treatment approach.

## 5. Conclusions

This meta-analysis revealed that higher NLR values, which serve as a potential indicator of adverse prognosis across several infectious diseases, represent a promising biomarker of the severity of FG and a prognostic indicator of eventual survival. Further studies are needed to cement the identified correlations and establish specific cutoff values to aid in decision-making. The identification of reliable biomarkers such as the NLR could significantly improve patient management and treatment outcomes in the clinical setting.

## Figures and Tables

**Table 1:** Characteristics of the studies included in the systematic review.

Author, Year	Country	Groups	N	Sex	Age (y)	DM (n)	Hypertension (n)	Outcome
Wijropranoto, 2023 [32]	Indonesia	Survivors	112	M: 103 | F: 9	50.4 ± 15	63	9	NLR (MD) NLR (Cutoff)
		Non-Survivors	23	M: 20 | F: 3	53.2 ± 14.7	11	3	
Shin, 2022 [33]	South Korea	Survivors	58	M: 53 | F: 5	58 ± 14	23	23	NLR (MD)
		Non-Survivors	27	M: 20 | F: 7	65.3 ± 15	8	9	
He, 2022 [34]	China	Survivors	13	M: 9 | F: 4	48.6 ± 13.6	4	NR	NLR (MD)
		Non-Survivors	5	M: 3 | F: 2	60.6 ± 10.4	3	NR	
Raizandha, 2022 [35]	Indonesia	Survivors	90	NR	49 ± 14.9	40/109	NR	NLR (Cutoff)
		Non-Survivors	19	NR	54 ± 13.8		NR	
Karaali, 2020 [36]	Turkey	Survivors	69	M: 58 | F: 31	53.9 ± 13.57	31	19	NLR (MD)
		Non-Survivors	20		67.6 ± 11.52	13	7	
Ozluerden, 2020 [37]	Turkey	Survivors	65	M: 60 | F: 5	50.02 ± 16.4	30	NR	NLR (MD)
		Non-Survivors	18	M: 13 | F: 5	68.28 ± 14.58	13	NR	
Pehlivanli, 2019 [38]	Turkey	Survivors	18	M: 15 | F: 3	63 ± 16.33	9	NR	NLR (MD)
		Non-Survivors	5	M: 4 | F: 1	78 ± 10.83	2	NR	
Demir, 2018 [39]	Turkey	Survivors	68	M: 46 | F:22	58.3 ± 14.55	31	NR	NLR (MD)
		Non-Survivors	6	M: 3 | F: 3	49.66 ± 22.73	6	NR	
Wetterauer, 2018 [40]	Switzerland	Survivors	16	M: 16 | F: 0	57.71 ± 15.89	NR	NR	NLR (MD) NLR (Cutoff)
		Non-Survivors	2	M: 2 | F: 0	83 ± 14.93	NR	NR	
Kuzukdurmaz, 2017 [41]	Turkey	Survivors	31	NR	53.25 ± 16.07	15	NR	NLR (MD)
		Non-Survivors	7	NR	71.14 ± 12.5	2	NR	
Yim, 2016 [42]	South Korea	Survivors	36	M: 35 | F: 1	57.1 ± 14.4	10	NR	NLR (MD) NLR (Cutoff)
		Non-Survivors	26	M: 26| F: 0	56.2 ± 13	11	NR	
Bozkurt, 2015 [43]	Turkey	Survivors	30	NR	56 ± 12.8	22/33	10/33	NLR (Cutoff)
		Non-Survivors	3	NR	72.9 ± 7.3			

DM: Diabetes Mellitus; M: Male; F: Female; y: year; n: number; MD: Mean Difference; NR: Not reported.

## Data Availability

All data generated or analyzed during this study are included in this published article [and its supplementary information files].

## Supplementary Materials

The following supporting information can be downloaded at https://www.mdpi.com/article/10.3390/idr17030055/s1, SDC 1. Table S1: PRISMA Checklist; SDC 2. Table S2: ROBINS-I risk of bias assessment; SDC 3. Figure S1: Traffic light plot for risk of bias assessment of the comparative studies; SDC 4. Figure S2: NLR mean difference funnel plot for survivors vs nonsurvivors; SDC 5. Figure S3: Sensitivity analysis of the NLR mean difference. Funnel plot of survival vs nonsurviving patients; SDC 6. Figure S4: Sensitivity analysis excluding high-risk of bias studies on the NLR mean difference. Funnel plot of survival vs nonsurviving patients; SDC 7. Figure S5: Funnel plot for the meta-analysis of the studies using an NLR cut-off, assessing the FG mortality risk; SDC 8. Figure S6: Funnel plot for the meta-analysis of the studies using an NLR cutoff=8 assessing the FG mortality risk; SDC 9. Figure S7: Funnel plot for the meta-analysis of the studies using an NLR cutoff=10 assessing the FG mortality risk; SDC 10. Figure S8: Scatter lot of age‒NLR values for the survivor group indicating the absence of correlation between the 2 variables; SDC 11. Figure S9: Scatter lot of age‒NLR values for the non-survivor group indicating the absence of correlation between the 2 variables; SDC 12. Figure S10: Sensitivity Analysis excluding high-risk of bias studies on the NLR Mean Difference: Forest Plot Survivors vs. Non-Survivors; SDC 13. Figure S11: Subgroup Analysis based on country of study origin of the NLR Mean Difference: Forest Plot Survivors vs. Non-Survivors; SDC 14. Figure S12: Forest plot for the meta-analysis of the studies using an NLR cutoff=8 assessing the FG mortality risk; SDC 15. Figure S13: Forest plot for the meta-analysis of the studies using an NLR cutoff=10 assessing the FG mortality risk.

## Abbreviations

The following abbreviations are used in this manuscript:

FG	Fournier Gangrene
FGSI	Fournier Gangrene Severity Index
SFGSI	Simplified Fournier Gangrene Severity Index
LRINEC	Laboratory Risk Indicator For Necrotizing Fasciitis
NLR	Neutrophil-to-Lymphocyte Ratio
PRISMA	Preferred Reporting Items for Systematic Reviews and Meta-Analyses

## Appendix A

### Pubmed Search String

(“Fournier gangrene”[MeSH Terms] OR “Fournier Gangrene”[Title/Abstract]) AND (“prognosis”[MeSH Terms] OR “prognosis”[Title/Abstract] OR “prognostic factor”[MeSH Terms] OR “prognostic factor”[Title/Abstract] OR “NLR”[Title/Abstract] OR “neutrophil-to-lymphocyte ratio”[Title/Abstract] OR “neutrophil-to-lymphocyte ratio”[Title/Abstract] OR “neutrophil lymphocyte ratio”[Title/Abstract])
